# A novel infrared fluorescence method to identify regions of superficial microvenous reflux in patients with chronic venous disease

**DOI:** 10.1016/j.jvsv.2026.102448

**Published:** 2026-01-19

**Authors:** Gregory T. Jones, Kari Clifford, Geraldine B. Hill, Kate N. Thomas, Sarah Lesche, Jolanta Krysa

**Affiliations:** Department of Surgery and Critical Care, Faculty of Medicine, Dunedin, University of Otago, Dunedin, New Zealand

**Keywords:** Chronic venous disease, Microvasculature, Near infrared fluorescence, Varicose veins, Venous reflux

## Abstract

**Objective:**

Reflux within the superficial microvenous network may play a critical role in the development of skin changes associated with chronic venous insufficiency. This study aimed to extend previous ex vivo observations to determine the in vivo utility of near infrared fluorescence (NIRF) imaging to assess superficial venous reflux in the leg.

**Methods:**

A total of 28 limbs were examined in 17 participants. These included limbs with (CEAP C2, n = 6; C3, n = 1; and C4, n = 15) and without (CEAP C0, n = 6) venous disease. Indocyanine green (5 mL at 0.1 mg/mL) was infused via an (antegrade) cannula in the distal great saphenous vein and the medial leg imaged using NIRF. Venous reflux was assessed using the Valsalva maneuver, with or without superficial outflow obstruction (thigh cuff inflated to 50 mmHg).

**Results:**

Consistent with our previous ex vivo study, NIRF imaging visualized a wide range of different microvenous reflux patterns in vivo. These included focal and diffuse regions of fluorescence within the skin, the extent of which appeared to be associated with venous disease (CEAP C classification) severity. The observed reflux patterns also appeared to be functional correlates of perforator vein or saphenofemoral junctional incompetence.

**Conclusions:**

This preliminary in vivo study provides proof-of-principle observations suggesting a potential novel method for investigating microvenous reflux in superficial venous disease.

**Clinical Relevance:**

This study reports the first in vivo use of near-infrared fluorescence (NIRF) imaging with indocyanine green to assess superficial microvenous reflux within intact limbs. This preliminary data suggests that the extent and distribution of skin fluorescence may be associated with venous disease severity (CEAP Clinical classification). It also provides potential mechanistic insight, identifying reflux patterns that appear to be functional correlates of venous incompetence. This study suggests that NIRF imaging could provide a novel tool for investigating microvenous contributions to chronic venous disease and its skin manifestations.

**Article Highlights**

• **Type of research**: Prospective, single-center, observational study
• **Key Findings**: This study reports the first in vivo use of near-infrared fluorescence (NIRF) imaging with indocyanine green to assess superficial microvenous reflux. It confirmed that NIRF microvenous reflux patterns can also be visualized within intact limbs. This preliminary data suggests that the extent and distribution of skin fluorescence may be associated with venous disease severity (CEAP classification). It also provides potential mechanistic insight, identifying reflux patterns that appear to be functional correlates of perforator vein or saphenofemoral junctional incompetence.
• **Take Home Message:** These results suggests that NIRF imaging could provide a novel, noninvasive tool for investigating microvenous contributions to chronic venous disease and its skin manifestations.

Chronic venous disease (CVD) is a common condition^1^ associated with a significant global socioeconomic burden.^2^^,^^3^ Age, family history of varicose veins, deep venous thrombosis, obesity, and (large vessel) venous reflux are known to be risk factors for venous disease progression.^4^^,^^5^ Nevertheless, predicting those patients with CVD who will progress to more severe clinical manifestations, such as skin changes and ulceration, remains challenging.

The current recommendations from the European Society of Vascular Surgery state that duplex ultrasound should be the primary diagnostic test to identify the source and pattern of reflux.^4^ These guidelines are focused on the larger superficial and deep veins, which is appropriate given the importance of these vessels in the pathophysiology and management of CVD. There is, however, a growing awareness that ‘ascending incompetence’ may be a potential pathogenic contributor and that the ability to accurately assess superficial microvenous reflux may also be beneficial.^6^, ^7^, ^8^

In this regard, a series of previous ex vivo studies by our group suggested that valvular incompetence within small superficial veins of the leg could be a contributing factor in the progression of CVD.^9^^,^^10^ In these prior studies, retrograde venous corrosion casts were used to document valvular incompetence in superficial venous drainage networks. The presence of an incompetent series of superficial veins enabled reflux of the casting resin from the primary drainage vessel (eg, the great saphenous vein [GSV]) back to the microvenous network, forming the drainage component of the microvascular circulation of the skin. Following the identification of these incompetent superficial venous networks, we then developed a method that could potentially identify such regions in vivo. This involved imaging near-infrared fluorescence (NIRF) of indocyanine green (ICG) within the distal GSV, injected via the same site used previously to produce retrograde venous corrosion casts of the medial calf.^9^ ICG is a fluorescent dye that is retained within the vascular space, making it suitable for evaluating tissue perfusion.^11^ Any ICG transiting from the GSV into the overlying dermal microvenous network was imaged by a detector positioned over the medial calf region and was considered to be indicative of reflux. It is important to note that this NIRF-based method was developed using ex vivo amputated limbs.^10^ A range of NIRF-detected reflux patterns, from absent to extensive, were detected, and these appeared to correlate to disease severity.^10^ However, a substantial limitation of these previous investigations was the absence of intact physiology, with a clear need to determine if reflux within a live limb is detectable with this technique.

This study therefore aimed to translate our ex vivo developed and validated NIRF method into a means of detecting the presence of superficial microvenous reflux in a clinically relevant in vivo setting.

## Methods

This prospective observational study examined 28 limbs in 17 participants. Participants consisted of patients with venous disease who were undergoing clinical assessment by the Vascular Assessment Laboratory (Department of Surgery and Critical Care - Dunedin, University of Otago) or healthy community volunteers. Each limb was assessed by an experienced vascular surgeon and categorized using the CEAP Clinical (C) classification.^12^ All participants provided written informed consent, and the study was approved by the New Zealand Health and Disability Ethics Committee (reference 2022 EXP 12,652). Inclusion criteria were age between 18 and 95 years and the ability to provide informed consent. Exclusion criteria were known history of peripheral artery disease (PAD) or abnormal ankle-brachial pressure index (greater than 1.1 or less than 0.9) or history of venous ulceration (CEAP C5 or CEAP C6) to avoid risk of any hypersensitivity reactions^13^ that may have the potential to exacerbate further trophic skin changes and/or ulceration.

A venous ultrasound assessment of the medial calf/gaiter region of each limb was conducted prior to performing NIRF imaging. All limbs with CEAP C2 to 4 had also undergone a comprehensive limb assessment within the previous 3 years (see Supplementary Fig, *A* and *B*, online only). The competence (tested via manual augmentation) of the GSV and the associated calf perforators was documented.

To improve participant comfort and aid consistent positioning of the NIRF imaging head, participants were examined in a supine position (3^o^-4^o^ head-up tilt). The distal GSV was cannulated (20-gauge cannula and side flush port connector) just above the medial malleolus, to provide an access site. This process was conducted, under ultrasound guidance, by a qualified vascular surgeon. Immediately prior to dye infusion, an external pressure cuff was placed around the distal thigh and inflated to 50 mmHg. The NIRF imaging head (Novadaq SPY Elite system, Stryker Corporation) was positioned over the medial leg and focused on the skin surface. The distal border of the imaging field was placed 2 cm above the cannulation site, and the proximal border was indicated on the skin with a small black pen mark. The size of the NIRF imaging field was approximately 22 × 16.5 cm. The limb was continuously imaged by NIRF, with an illumination and detection wavelength of 805 nm, throughout the study, with a typical study lasting 5 to 6 minutes. Bright field images were captured, using the inbuilt imaging head camera, pre and post NIRF imaging.

Indocyanine green (5 mL at 0.1 mg/mL, Verdye, Diagnostic Green GmbH) was then infused antegrade via the GSV cannula, followed 20 seconds later by a 10 mL saline flush. After the flush, participants were asked to perform three Valsalva maneuvers (as a reflux challenge) with 30-second intervening rest periods. Most Valsalva maneuvers were of 8- to 10-second duration. The thigh cuff was then deflated, and a further two Valsalva maneuvers were performed. Manual augmentations, such as calf or foot compressions, were not used to avoid substantial movements of the limb within the imaging field.

## Results

### Cohort demographics

The age, sex, and limb CEAP Clinical classification of the seventeen study participants are shown in the Table. A total of 28 limbs from 17 participants were examined. These included limbs with (CEAP C2, n = 7; C3, n = 1; and C4, n = 14) and without (CEAP C0, n = 6) venous disease.

**Table:** Study participant demographics

Case # and limb (L/R)	Sex	Age, years	CEAP C classification
1 R	M	54	0
1 L			0
2 R	F	44	0
2 L			0
3 R	M	22	0
3 L			0
4 R	F	31	2
4 L			2
5 R	F	55	2
5 L			2
6 R	F	35	2
6 L			4
7 R	M	64	4
7 L			2
8 R	F	53	4
8 L			2
9 R	F	66	3
10 R	M	79	4
11 R	F	38	4
12 R	F	72	4
13 R	M	55	4
14 R	M	77	4
14 L			4
15 R	M	57	4
15 L			4
16 R	F	75	4
16 L			4
17 R	F	68	4

*F,* Female; *L,* left; *M,* male; *R,* right.

#### Detecting patterns that were potentially consistent with microvenous reflux

A primary objective of this study was to determine if ICG infusion within intact (in vivo) limbs could result in NIRF patterns consistent with our previous ex vivo microvenous reflux observations. In all but one limb, some degree of NIRF was observed. Case 11 was a right CEAP C4 limb from a 38-year-old female with ICG fluorescence visible within the syringe and catheter prior to infusion. The ICG and flush saline flowed readily into the GSV at the initiation of the NIRF imaging protocol; however, no NIRF was detectable within the limb itself.

**Fig 1:**
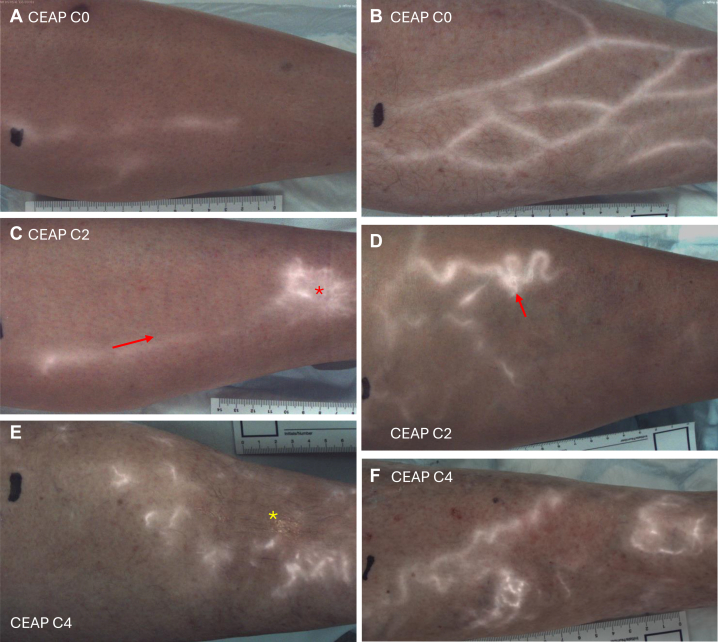
Near-infrared fluorescence (NIRF) venous reflux patterns in different chronic venous disease (CVD) clinical classifications. **(A and B)** CEAP C0, **(A)** Case#2L, female 44 years, **(B)** Case#1L, male 54 years. Note the filling of numerous superficial venous tributaries; however, in both A and B there was no NIRF movement on Valsalva maneuver and no filling of smaller (<2 mm) veins. **(C and D)** CEAP C2, **(C)** Case#4L, female 31 years. A large stellate region of fluorescence (*asterisk*), with dye observed entering the region from a superficial accessory vein (*arrow*). Of note, this region was spontaneously reported as an area of irritation by the participant prior to the imaging being conducted. **(D)** Case#8 L, female 53 years. Reflux filling of a cluster of small veins (<1 mm diameter) was observed originating from a serpentine (4 mm) vein (*arrow*). **(E and F)** CEAP C4, **(E)** Case#12R, female 72 years. Numerous discrete regions of venous reflux surround a region of lipodermatosclerosis (*yellow asterisk*). **(F)** Case#14 L, male 77 years. See also Supplementary Videos 2-7 (online only).

In the remaining 27 limbs, a range of fluorescence patterns were observed, from filling of superficial accessory/tributary veins (3-4 mm diameter) (Fig 1, *A* and *B*) to extensive bright fluorescence within stellate (radiating from a central point) clusters of small (600-900 μm) veins (Fig 1, *C-F*). Of note, and consistent with our previous ex vivo studies, fluorescence was not typically visible within the GSV itself but was first observed in more superficial tributary veins before extending into smaller, but distinct, microvenous channels, and finally forming a more diffuse fluorescence ‘blush.’

**Fig 2:**
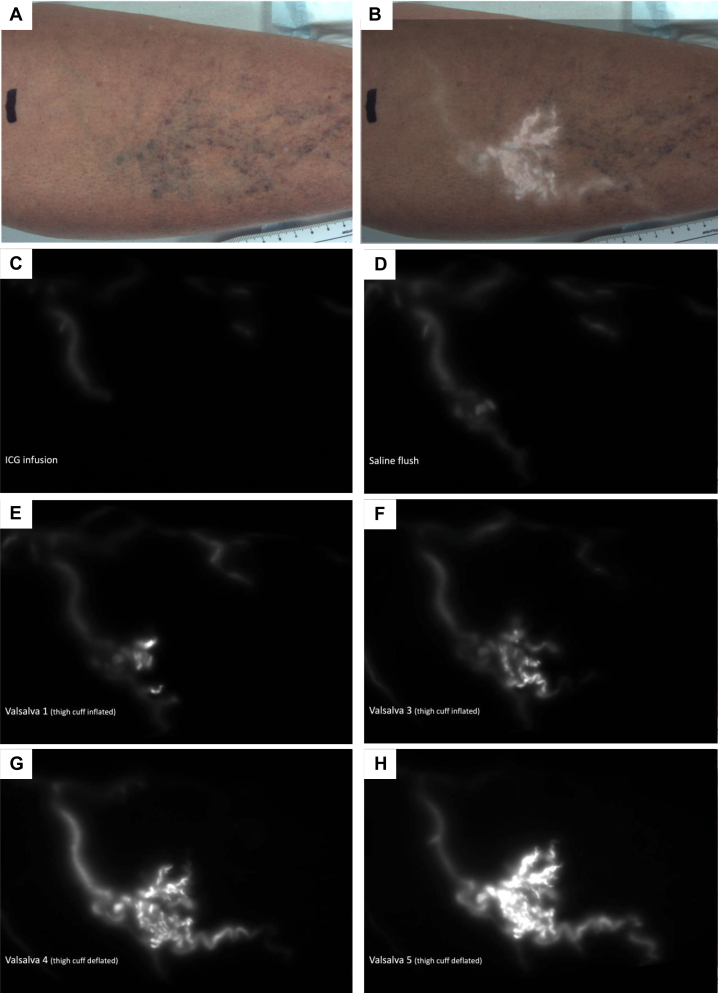
Augmentation of near-infrared fluorescence (NIRF) venous reflux. Representative frame captures (from Supplementary Video 1, online only) of each step in the NIRF imaging protocol from a CEAP C2 (Case#5R, female 55 years). **(A)** Bright field image. **(B)** Brightfield merged with NIRF (panel H). **(C)** Ten seconds after 5-mL indocyanine green (ICG) infusion, **(D)** 10 seconds after 10-mL saline flush. Peak fluorescence following the **(E)** first and **(F)** third Valsalva augmentations (with thigh cuff inflated to 50 mmHg). **(G and H)** Peak fluorescence in two subsequent Valsalva maneuvers with cuff deflated. Note the progressive additive retrograde filling of a focal venous network with each phase, and the more pronounced effect of Valsalva maneuver (in this participant) after thigh cuff deflation. See Supplementary Video 1 (online only).

A representative series of images of a single limb taken at each step of the NIRF protocol is shown in Fig 2 (see also matching Supplementary Video 1, online only). Typically, minimal and faint fluorescence was observed in tributary veins (∼4 mm diameter) in association with the initial 5-mL ICG dye infusion (Fig 2, *C*). The subsequent 10-mL saline flush enhanced this fluorescence and extended it further along the venous network (Fig 2, *D*). The extent of subsequent filling appeared to be associated with the limb’s clinical classification. In all of the (venous disease-free) CEAP C0 limbs, NIRF remained limited to the (3-4 mm diameter) venous tributaries (Fig 1, *A* and *B*). In CEAP C2 limbs, one or two focal clusters of smaller veins, arising from associated fluorescing tributaries, were typically observed (Fig 1, *C* and *D*; Supplementary Fig, *C*, online only). In CEAP C4 limbs, multiple microvenous NIRF regions were observed, along with areas of faint diffuse fluorescence (Fig 1, *E* and *F*). Regions of diffuse fluorescence, which our previous work had shown to be associated with dermal capillary filling, were best observed in the isolated NIRF images rather than those merged with the brightfield images of the limb (Supplementary Fig, *A*, *B*, *D-F*, online only).

#### The effect of a reflux challenge on NIRF in the superficial microvenous network

After the ICG infusion and saline flush, Valsalva maneuvers were performed as a venous reflux challenge (Supplementary Videos 2-7, online only). The presence of a thigh cuff, inflated to 50 mmHg, limited superficial venous outflow during the initial ICG and saline infusion. Cuff inflation during the first three Valsalva maneuvers was intended to test for segmental deep to superficial reflux within the calf (eg via perforating veins). Valsalva maneuvers following thigh cuff deflation was intended to test for the potential effects of reflux through incompetent proximal superficial veins fed by the saphenofemoral junction and/or thigh perforator veins.

In all venous disease-free CEAP C0 limbs, Valsalva maneuvers had no substantial effect on NIRF dye movement within tributary veins (Supplementary Video 2, online only). In CEAP C2 to 4 participants, there was a clear, progressive, retrograde shift of NIRF into smaller vessels following each Valsalva maneuver (Fig 2, *E-H*). This effect appeared to be more pronounced in CEAP C4 limbs and, in some cases, the magnitude of effect appeared to be greater when the Valsalva maneuver was performed with the thigh cuff deflated (compare Fig 2, *E* and *F* with Fig 2, *G* and *H*). These differential effects were best appreciated within video sequences (see Supplementary Videos 6 and 7, online only).

Some participants had minor superficial skin abrasions and scarring, which were typically attributed to outdoor activities such as gardening. There was no apparent association between such sites of injury and NIRF (Supplementary Fig, *A* and *F*, online only).

Finally, reproducibility of observations was confirmed in two participants (Case #1L: CEAP C0 and Case #5L: CEAP C2) who underwent repeat NIRF assessments performed more than 6 months apart. The were no differences in the observed NIRF patterns (ie, specific vessels filled or response to Valsalva maneuvers) between the separate assessment periods.

## Discussion

In this study, we assessed the utility of NIRF as a method for identifying regions of superficial microvenous reflux in vivo. This was an extension of a previous ex vivo study, which compared NIRF with matching X-ray contrast venography and vascular corrosion casting.^10^ The observed ex vivo NIRF patterns appeared to be consistent with both valvular incompetent reflux and venous disease severity^10^; however, given that these studies were performed in amputated limbs, the translational potential of these observations were substantially limited by the absence of intact physiology. The ICG dose used in this study was specifically determined in our previous ex vivo studies showing that a 5-mL infusion of 0.1 mg/mL resulted in optimal small vessel imaging in the context of this specific application.^10^ ICG has been shown to an effective method for assessing cutaneous vascular perfusion.^11^^,^^14^, ^15^, ^16^ In addition, ICG has an excellent safety profile, including a very low rate of hypersensitivity reactions.^17^^,^^18^ No hypersensitivity or other adverse reactions were observed in this study, noting that a total dose of only 0.5 mg ICG was administered, with 0.5 mg/kg being considered safe.^14^

In the current in vivo study, although NIRF was detected within tributary veins in CEAP C0 venous disease-free limbs, this was not responsive to the Valsalva maneuver. A key distinction between CEAP C0 limbs and those with documented superficial venous disease was the extension of the fluorescent dye beyond the large (3-4 mm diameter) tributary veins into smaller venous branches (<1 mm in diameter). The number and extent of regions suggestive of microvenous reflux appeared more pronounced in limbs with venous disease-associated skin changes (CEAP C4) than those with uncomplicated varicose veins (CEAP C2). Previous work has demonstrated that when valvular incompetence occurs in superficial veins three to four branch generations from the primary drainage vessel (so-called boundary valves), the likelihood and extent of microvenous and dermal capillary retrograde filling is substantially increased.^9^^,^^10^ Boundary valve incompetence was also suggested as a key contributor to the increased microvenous reflux observed in limbs with venous ulceration.^9^ We suggest that these current NIRF observations appear broadly consistent with this concept.

### Methods for assessing superficial venous reflux

The European Society of Vascular Surgery guidelines state that duplex ultrasound should be the primary diagnostic test to identify the source and pattern of venous reflux.^4^ Consequently, reflux is defined as “retrograde flow during ultrasound examination lasting more than 0.5 seconds in the superficial venous system, the deep femoral and calf veins, longer than 1 second in the common femoral, femoral vein, and popliteal vein, and longer than 0.35 seconds in perforating veins.^19^ A focus on aberrant physiology within larger superficial and deep veins is appropriate, given the importance of these vessels in the pathophysiology and management of CVD. However, it should also be recognized that it is more difficult to assess microvenous reflux, and as such, this aspect of venous physiology has been afforded less attention. Given that the pathophysiology of (micro)venous disease-related skin changes are known to be distinct from that of larger veins,^20^ the ability to accurately assess superficial microvenous reflux could clearly be beneficial. Some specific ultrasound signal filtering modalities for the in vivo detection of microvenous reflux have been developed; however, these tend to be strongly influenced by motion artifact and are limited to detection of flow within the ultrasound imaging field.^21^ In contrast, NIRF can simultaneously image the entire medial calf and identify distinct focal regions of superficial reflux.

### Study limitations

This study, being preliminary in nature, has several limitations. The comparisons were largely qualitative and were not designed to determine if NIRF was significantly different between venous disease clinical classifications. Although there were apparent qualitative differences between limbs that were venous disease-free (CEAP C0), uncomplicated varicose vein (C2), or those with venous disease-related skin changes (C3 or 4), further work, including a larger sample size, blinded image interpretation, inter-/intra-observer reliability testing, and a standardized scoring system, is required to determine the prognostic utility of the NIRF-detected microvenous reflux with regard to chronic superficial venous disease progression.

Given that this was a novel proof of principle study, C5 to 6 limbs are excluded to avoid risk of any hypersensitivity reactions^13^ that may have the potential to exacerbate further trophic skin changes and/or ulceration. Moreover, we would suggest that there is no clinical relevance in performing this test in such a group as they have already presented with skin breakdown, making a NIRF-informed disease progression risk score irrelevant in the context of appropriate patient management.

To improve participant comfort and aid consistent positioning of the NIRF imaging head, participants were examined in a near supine position. With regards to the use of the Valsalva maneuver, optimal detection of superficial lower limb reflux has been suggested when performed while the patient is in the 15° reverse Trendelenburg position.^22^ Nevertheless, Valsalva maneuver performed in a supine position was still able to elicit a reflux response that appeared consistent with ultrasound confirmed patterns of segmental venous incompetence. An example of this is shown in Case#16R (Supplementary Fig, *A*, online only and the matching Supplementary Video 6, online only). In this case, duplex ultrasound confirmed that the proximal GSV was incompetent, whereas the distal calf segment was competent (Supplementary Fig, *A*, online only, panel vii). Consistent with this, NIRF findings indicated a blunted response to Valsalva maneuver when the thigh cuff was inflated (thereby blocking proximal GSV reflux) and a pronounced effect on dye reflux when the cuff was deflated.

### Prospective utility of NIRF-detected reflux

This was a proof-of-principle feasibility study, aiming to confirm the translational potential of a NIRF-based technique to detect superficial microvenous reflux. Our in vivo observations support this contention, with our preliminary evidence demonstrating NIRF detection of microvenous reflux in patients with CVD.

Microvenous reflux and associated dermal capillary distension^23^, ^24^, ^25^ have been suggested as precursors to more severe forms of CVD^23^; therefore the ability to reliably identify such regions could provide several clinical and diagnostic benefits. Identifying microvenous reflux early may allow for more proactive management, potentially preventing progression to more severe symptoms, particularly those related to venous disease-related skin changes and ulceration. Other benefits could include the identification of incompetent tributaries suitable for targeted interventions, such as sclerotherapy, and thereby potentially avoiding unnecessary procedures on larger veins. Microvenous reflux might play a role in highlighting regions with greater risk of recurrence following CVD treatment. The use of a NIRF-based scoring system could also contribute to more refined diagnostic criteria and risk stratification for CVD.

## Conclusions

NIRF enabled the in vivo identification of focal regions of superficial microvenous reflux. This proof of principle study demonstrates the potential clinical utility for NIRF as a method for investigating superficial venous disease, particularly its influence on the microvenous circulation. Future, statistically powered, assessor-blinded, longitudinal follow-up studies will be required to determine if NIRF identified microvenous reflux has any venous disease progression prognostic clinical utility (eg, whether it can predict those with CEAP C2 that are at increased risk progression to venous skin changes or ulceration) beyond that of the current standard diagnostic tools, such as duplex ultrasound.

## Author Contributions

Conception and design: GJ, JK

Analysis and interpretation: GJ, KC, JK

Data collection: GH, KT, SL

Writing the article: GJ

Critical revision of the article: KC, GH, KT, SL, JK

Final approval of the article: GJ, KC, GH, KT, SL, JK

Statistical analysis: Not applicable

Obtained funding: GJ, JK

Overall responsibility: GJ

## Funding

This work was supported by a 10.13039/501100009477Lottery Health (NZ) Grant and the H.S. and J.C. Anderson Trust.

## Disclosures

None.

## Appendix (online only)

Each sequence begins with the brightfield appearance of the limb, followed by the real-time near-infrared fluorescence imaging (NIRF) video and followed by a merged NIRF/brightfield image.

Video 1 NIRF during protocol steps CEAP C2 (Case #5R, Female, 55 years). This video includes sequences for each step of the NIRF imaging protocol: initial (5 mL) indocyanine green (ICG) infusion, followed by (10 mL) saline flush of the infusion catheter. This was followed by a series of deep system augmentations (Valsalva maneuvers) with, then without, a 50-mmHg thigh cuff. Cuff inflation during Valsalva augmentations was intended to test for segmental deep to superficial reflux within the calf (via perforating veins). Valsalva following thigh cuff deflation was intended to test for the potential effects of reflux through incompetent proximal superficial veins. In this example, note the progressive retrograde movement of NIRF during successive Valsalva (cuff inflated). This was much more pronounced during subsequent Valsalva performed when the thigh cuff was deflated. This suggests are greater superficial venous reflux potential via the proximal great saphenous vein (GSV) than from the segmental calf region. See also Fig 2.

Video 2 Valsalva CEAP C0 (Case #1L, Male, 54 years). This video sequence shows NIRF movement during a Valsalva (thigh cuff inflated). Although the several tributary veins filled with dye during the initial infusion and saline flush, Valsalva had no substantial effect on NIRF dye movement within tributary veins. See also Fig 1, *B*.

Video 3 Valsalva CEAP C2 (Case #4L, Female, 31 years). This video sequence shows NIRF movement during a second Valsalva (thigh cuff inflated). The *red circle* in the brightfield image indicates a region that was spontaneously reported as an area of irritation by the participant prior to the NIRF imaging being conducted. This area became brightly NIRF-positive on first Valsalva. On second Valsalva, the large stellate region of fluorescence (*yellow circle*) was displaced by flow that appeared to be refluxing through the associated tributary vein. See also Fig 1, *C*.

Video 4 Valsalva CEAP C2 (Case #8L, Female, 53 years). This video sequence shows Valsalva (thigh cuff deflated)-driven reflux filling of a cluster of small (<1 mm diameter) venous network originating from a larger serpentine (4 mm) vein. Consistent with our previous ex vivo studies, note the brighter fluorescence of the smaller veins (likely due to their more superficial location compared with the underlying feeding vein). See also Fig 1, *D*.

Video 5 Valsalva CEAP C4 (Case #12R, Female, 72 years). In this video sequence, numerous discrete regions of NIRF venous reflux, surrounding a region of lipodermatosclerosis, are observed during a second Valsalva (thigh cuff inflated). See also Fig 1, *E*.

Video 6 Valsalva ×2 CEAP C4 (Case #16R, Female, 75 years). In this video sequence, the effect of Valsalva with or without the thigh cuff inflated is shown. With the cuff inflated, there is only subtle movement of the NIRF during Valsalva. In contrast, more extensive augmentation associated movement is observed when the thigh cuff has been deflated (see also Supplementary Fig, *E*, online only). This limb had extensive diffuse fluorescence such that the outline of the limb could be seen in the NIRF imaging mode.

Video 7 Valsalva ×2 CEAP C4 (Case #17R, Female, 68 years). This video also shows the effect of Valsalva with or without the thigh cuff inflated. In contrast to that shown in Supplementary Video 5 (online only), the NIRF demonstrates retrograde displacement regardless of cuff inflation, consistent with ultrasound finding of both proximal and segmental (calf perforator vein) reflux. See also Supplementary Fig, *F* (online only).
