# Supplementary material for: A novel infrared fluorescence method to identify regions of superficial microvenous reflux in patients with chronic venous disease
Source: J Vasc Surg Venous Lymphat Disord. 2026 Jan 19;14(3):102448. doi: 10.1016/j.jvsv.2026.102448 (PMC12925454; doi:10.1016/j.jvsv.2026.102448)
Supplement: Supplementary Figures [file mmc8.docx]

**Supplementary Figures**

**Valsalva, with and without thigh cuff inflation**

**Supplementary Figure A. CEAP C4 (Case#16R, Female, 75 years)**

**Supplementary Figure B. CEAP C4 (Case#17R, Female, 68 years)**

**Bright field, NIRF and Combined Images**

**Supplementary Figure C. CEAP C2 (Case#8L, Female, 53 years)**

**Supplementary Figure D. CEAP C4 (Case#14L, Male, 77 years)**

**Supplementary Figure E. CEAP C4 (Case#15L, Male, 57 years)**

**Supplementary Figure F. CEAP C4, (Case#16L, Female, 75 years)**

**Supplementary Figure A. CEAP C4 (****Case#16R, Female, 75 years)**

i

ii

iii

iv

v

vi

vii

In this limb CEAP C4 limb, (i) NIRF became extensively visible on initial (5mL) ICG, (ii) then even more pronounced during the 10mL saline infusion. (iii) Only subtle NIRF movement was observed when Valsalva was performed with the thigh cuff inflated. In contrast, after the thigh cuff was deflated there was a noticeably more pronounced movement of NIRF into the microvenous and capillary network during Valsalva. (v) mid Valsalva (vi) late Valsalva (cuff deflated). As shown in supplementary video 6.

(vii) Ultrasound reporting suggested that the primary source of superficial reflux was via the proximal GSV. Red arrows denote detection of incompetent flow, while blue show segments with only normal anterograde flow.

**Supplementary Figure 1B. CEAP C4 (Case#17R, Female, 68 years)**

i

iv

ii

iii

The NIRF frames captured after (ii) the third (thigh cuff still inflated) and (iii) fourth (thigh cuff still deflated) Valsalva manoeuvre. In both instances the NIRF noticeably shifted further into the microvenous network (see supplementary video 7). (iv) This is consistent with the ultrasound findings which documented both proximal GSV and calf perforator vein incompetence. Red arrows denoted detection of incompetent flow, while blue show segments with only normal anterograde flow.

**Supplementary Figure C. CEAP C2 (Case#8L, Female, 53 years)**

i

ii

iii

This is the same limb as shown in Figure 1D of the main paper but shown as separate bright field and NIRF images. (B) The isolated NIRF image make it easier to see the single stellate (arrow; radiating from a central point) microvenous cluster arising from serpentine tributary. The filling of this microvenous cluster can be observed being driven by a deep system augmentation (Valsalva manoeuvre) in supplementary video 4.

**Supplementary Figure D. CEAP C4 (Case#14L, Male, 77 years)**

i

*

*

ii

iii

The NIRF frame (ii) was captured after the third Valsalva (thigh cuff still inflated).

The asterisks denote faint diffuse fluorescence (no distinct vessels being resolved). Such regions appear consistent with the capillary filling previously observed in our *ex vivo* NIRF and resin vascular casts studies (*Eur J Vasc Endovasc Surg* 2022;**64**: 377-86).

The extensive NIRF filling pattern, which showed progressive filling on repeated Valsalva, was observed with the thigh cuff inflated, which suggesting the presence of distal segmental (calf) deep to superficial incompetence. This conclusion was supported by duplex ultrasound reporting incompetent calf perforator veins.

**Supplementary Figure E. CEAP C4 (Case#15L, Male, 57 years)**

i

ii

iii

Another example of the extensive NIRF filling pattern typically observed in CEAP C4 limbs. Again the NIRF filling was progressively responsive to repeated Valsalva. Note the intensity of fluorescence such that the skin marker was visible by NIRF (left hand side of panel ii).

**Supplementary Figure F. CEAP C4, (Case#16L, Female, 75 years)**

i

ii

iii

Multiple, brightly fluorescent, stellate (arrows; radiating from a central point) clusters arising from fainter tributaries. In addition, this participant had several sites of skin injury (self-reported as being due to their gardening activities) which did not show NIRF despite extensive fluorescence in the surround adjacent tissues.
